# Design and Application of Highly Efficient Flame Retardants for Polycarbonate Combining the Advantages of Cyclotriphosphazene and Silicone Oil

**DOI:** 10.3390/polym11071155

**Published:** 2019-07-05

**Authors:** Jicheng Jiang, Yanbin Wang, Zhonglin Luo, Tianyi Qi, Yihui Qiao, Menghao Zou, Biaobing Wang

**Affiliations:** Jiangsu Key Laboratory of Environmentally Friendly Polymeric Materials, School of Materials Science and Engineering, Jiangsu Collaborative Innovation Center of Photovolatic Science and Engineering, Changzhou University, Changzhou 213164, Jiangsu, China

**Keywords:** triphosphazene, flame retardant, silicone oil, polycarbonate

## Abstract

A novel flame retardant (HSPCTP) was successfully designed and incorporated into a polycarbonate (PC) matrix. Combining the advantages of cyclotriphosphazene and silicone oil, PC/HSPCTP composites passed UL-94 V-0 rating testing with only 3 wt% HSPCTP, and their LOI value increased from 25.0% to 28.4%. The findings showed that HSPCTP exhibits both gas-phase and solid-phase flame-retardant effects. Furthermore, the incorporation of HSPCTP into PC could suppress the release of smoke. Finally, the flame-retardant mechanism is discussed in depth.

## 1. Introduction

Due to its strong impact resistance, heat resistance, and dimensional stability, polycarbonate (PC) has been widely used in fields such as automobiles, office equipment, packaging, construction, sports equipment and health care industries. Although pure PC resin has a certain degree of flame retardancy (25% of LOI value and UL-94 V-2 rating), it is still not suitable in harsh applications requiring a more stringent flame-retardant rating. Many studies have been carried out on the improvement of the flame retardancy of PC in recent decades, and various kinds of flame retardants (including halogenated organic compounds, organophosphates, and silicon-containing compounds) have been developed [1,2,3,4,5,6,7,8,9,10,11,12,13]. For example, halogen-containing flame retardants are known to be highly efficient for PC. However, they release toxic and corrosive substances during their decomposition and cause environmental problems. Therefore, it is necessary to develop a halogen-free and environment-friendly flame retardant to improve the flame retardancy of PC.

Cyclotriphosphazene derivatives are important types of inorganic materials containing alternate phosphorus and nitrogen atoms in their cyclic skeletons [14,15,16]. Moreover, hexachlorocyclotriphosphazene (HCCP) is often used as a starting material to prepare cyclotriphosphazene derivatives due to the high reactivity of the Cl atom. Varieties of derivatives have been synthesized by the substitution reaction of Cl with organic nucleophiles (alkyl-CH_3_, alkoxy-OCH_3_, phenoxy-OPh, amine-NH_2_, etc.) [11,17,18,19,20]. The cyclotriphosphazene derivatives display both inorganic properties and organic properties, such as high temperature resistance, flame retardancy and self-extinguishing properties [21,22,23,24]. A phosphorus/nitrogen-containing compound based on maleimide and cyclotriphosphazene was synthesized by Yang and used as the flame retardancy for epoxy resin [25]. An LOI value of 36.5% and a UL94 V-0 rating were achieved with a high loading level of a 17 wt% flame retardant. Cao synthesized hexakis (4-nitrophenoxy) cyclotriphosphazene (HNTP) and combined it with ammonium polyphosphate to improve the intumescent flammability and thermal properties of acrylonitrile-butadiene-styrene copolymer (ABS). The LOI value of 25.6% and UL-94 V-0 rating were obtained in the case of ABS (70 wt%) /HNTP (15 wt%) /APP (15 wt%) [26]. Notably, although the flame-retardant grade of these composites reached V-0 rating, the weight ratios of the flame retardants were too high, which worsens the mechanical performance.

On the other hand, silicon-containing compounds have been considered as a kind of high-efficient flame retardants [27,28]. For example, Song synthesized carboxyl-containing polysiloxane [29] and incorporated it into PC to improve the flame-retardant abilities. The LOI value of the composites was 38.5% and the sample passed UL-94 V-0 testing even when the content of flame-retardant was as low as 1.0 wt%. Unfortunately, the use of platinum dioxide catalyst (PtO_2_) increased the cost of reaction and the difficulty of post-treatment.

Therefore, it is necessary to combine the excellent flame retardancy of cyclotriphosphazene with silicone oil. However, silicon-containing cyclotriphosphazene flame retardants have scarcely been reported. He et.al [30] employed hydroxyl silicone oil to replace chlorine on HCCP (SCP), however, the chlorine was not completely replaced. Furthermore, a relatively larger amount of flame retardant (7.5%) was required to achieve the UL94 V-0 flame-retardant rating for PC materials. 

In this study, a novel silicon-containing cyclotriphosphazene compound was designed and prepared through the substitution of hexachlorocyclotriphosphazene with dihydroxypropyl silicone oil and sodium phenoxide. It was found that chlorine atoms almost disappeared. Moreover, PC material reached the UL94 V-0 flame-retardant grade, with an amount of only 3 wt% of flame retardant. In addition, the flame-retardant mechanism was investigated deeply by thermogravimetric analysis/infrared spectrometry (TG-IR), scanning electron microscopy (SEM), and cone calorimeter tests.

## 2. Materials and Methods

### 2.1. Materials

Hexachlorocyclotriphosphazene (HCCP) was pursed from the Zibo Blueprint Chemical Co. Ltd. (Zibo, China). Dihydroxypropyl silicone oil (WSS-140, hydroxyl value 1.5%) was purchased from the Wushi New Material Co. Ltd. (Hangzhou, China). Phenol, Sodium (Na), Triethylamine (TEA), N-hexane, Tetrahydrofuran, Benzophenone, talcum powder, and polytetrafluoroethylene (PTFE) were purchased from the Jiangsu Qiangsheng Functional Chemical Co. Ltd. (Nantong, China). Dichloromethane was purchased from the Sinopharm Group Chemical Reagent Co. Ltd. (Shanghai, China). Polycarbonate was purchased from Bayer AG (Leverkusen, Germany).

### 2.2. Synthesis of HSPCTP

**Scheme 1:**
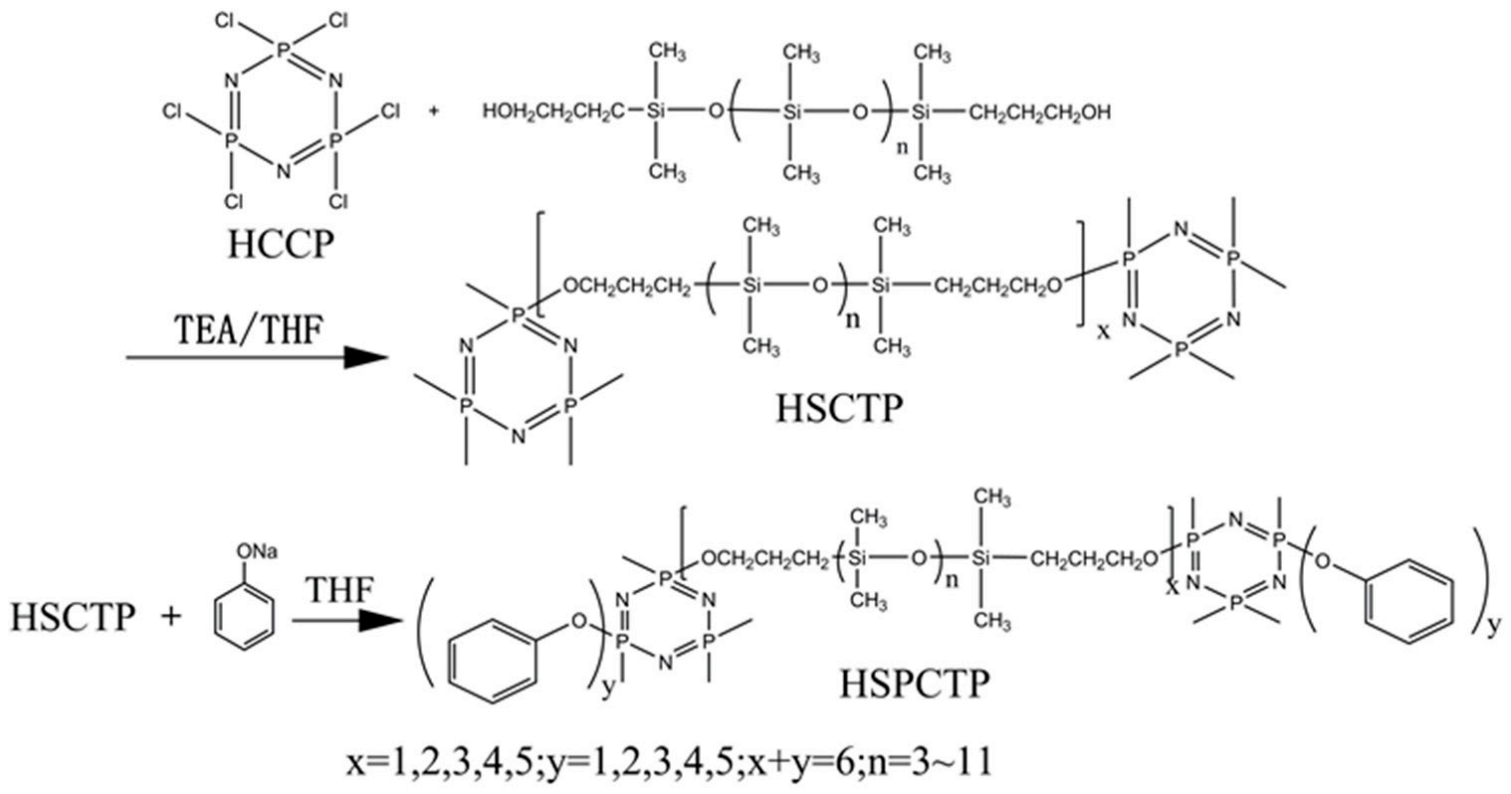
The synthetic route of HSPCTP.

The target product (HSPCTP) was prepared according to the following two steps and the synthetic routes are shown in Scheme 1.

Firstly, 3.0 g (8.63 mmol) hexachlorocyclotriphosphazene (HCCP), 16.40 g (8.63 mmol) bihydroxypropyl silicone oil and 10 mL dry tetrahydrofuran (THF) were added at 25 °C to a 250 mL four-necked flask equipped with a mechanical stirrer, a reflux condenser and a nitrogen inlet. Then the reaction temperature was raised to 70 °C, and 1.75 g (17.26 mmol) triethylamine was slowly added to the four-necked flask under a nitrogen atmosphere. After completion of the drop-wise addition, the reaction continued for another 24 h to obtain a yellow oily product, and was then filtered under reduced pressure to remove triethylamine salt. Finally, the lower liquid was separated and then the solvent was removed by rotary evaporation. An amount of 15.93 g HSCPT was obtained with a yield of 84.82%.

Anhydrous THF (20 mL) and 1.19 g (51.7 mmol) of sodium swarf were placed in a three-necked flask, and 4.87 g (51.7 mmol) of phenol was slowly added at room temperature. The reaction continued for 5–6 h. The sodium phenolate solution was successfully prepared.

The obtained intermediate product HSCPT and 10 mL dry THF were added to a four-necked flask equipped with a mechanical stirrer, a reflux condenser and a nitrogen inlet. After, the HSCPT was completely dissolved in dry THF and stirred. The reaction temperature was raised to 70 °C and the prepared sodium phenolate solution (5.00 g, 43.15 mmol) was slowly added to the four-necked flask under a nitrogen atmosphere. After completion of the addition, the reaction continued for 24 h to obtain a pale yellow viscous liquid product. The reaction mixture was concentrated by rotary evaporation to remove excess solvent and achieve a thick liquid. Then, the liquid was dissolved with the proper amount of methylene chloride and washed with plenty of deionized water 3–4 times until the water layer became clear. Afterwards, the lower liquid was separated and the solvent was removed by rotary evaporation. The liquid product was placed in a vacuum oven at 60 °C for 12 h. An amount of 17.35 g HSPCTP was obtained, with a yield of 84.65%.

### 2.3. Preparation of Flame-Retardant PC Composites

A series of PC/HCPTP composites were prepared by melt blending varying contents of HCPTP from 0 to 5 wt%. PC, talcum powder and polytetrafluoroethylene (PTFE) were placed in a vacuum oven and dried at 100 °C for 12 h before blending. Then, PC, talc, polytetrafluoroethylene (PTFE) and cyclotriphosphazene derivatives were added to the internal mixer and blended at 250 °C for 4 min. Then, the mixture was poured into the prepared molds. Thereafter, all samples were cooled slowly at room temperature in order to avoid cracking, and then the performance testing was carried out.

### 2.4. Characterization

The Fourier transform infrared (FTIR) spectra were investigated by a Nicolet 6700 FTIR (Madison, WI, USA) instrument at room temperature. The samples were mixed with KBr pellets and scanned 32 times over a spectral range of 4000–400 cm^−1^ with a resolution of 4 cm^−1^.

The ^29^Si NMR and ^31^P NMR spectra were conducted on a Bruker AV II- 400 MHz (Billerica, MA, USA) at room temperature, using DMSO-d6 as the solvent.

Elemental analysis spectra were investigated by a PerkinElmer EA 2400 II (Waltham, MA, USA) at room temperature in a vacuum environment.

To detect volatile pyrolysis products, a PerkinElmer TGA thermogravimetric analyzer was coupled to a PerkinElmer Fourier-transform infrared spectrometer (Waltham, MA, USA). Each sample was placed in an alumina crucible and heated from 50 to 700 °C at a heating rate of 20 °C/min under an N_2_ atmosphere. The thermogravimetric analyzer and the FTIR spectrometer were connected by quartz capillary at 230 °C.

The field-emission scanning electronic microscopy (FESEM), observed on an Hitachi SU8010 (Tokyo, Japan), was used to investigate the sectional morphology of the brittle fracture surface of flame-retardant PC composites and the surface of char residue of the flame-retardant PC composites after the vertical burning test was investigated by an Hitachi S3400 N (Tokyo, Japan). The surface of the particles and the char residue were sputter-coated with a conductive gold layer before observation. The EDS result of the residual char was measured by an energy-dispersive X-ray spectrometer.

Limiting oxygen indexes (LOI) were measured by an LOI analyzer (JF-3, Jiangning Co. Ltd., Nanjing, China) with sheet dimensions of 130 mm × 6.5 mm × 1.8 mm, according to GB/T 2406-93 standard. The vertical burning test (UL-94) of each sample was evaluated on a CZF-5-type instrument (Shine Ray Instrument Co. Ltd., Nanjing, China) with sheet dimensions of 130 mm × 13 mm × 1.8 mm according to ASTMD3801. An average of at least five replicas was used for each sample.

The fire behavior of the flame-retardant PC composites was evaluated via a cone calorimeter device (Fire Testing Technology, East Grinstead, UK) according to ISO 5660-1. The foursquare samples with the dimension of 100 mm × 100 mm × 3 mm were exposed to a radiant cone at a heat flux of 35 kW/m^2^. In both micro-combustion calorimetry and cone calorimetry tests, the data we obtained from the two parallel tests were close to each other (±5%).

## 3. Results

### 3.1. Characterization of Chemical Structure

#### 3.1.1. Fourier Transform Infrared (FTIR) Analysis

**Figure 1:**
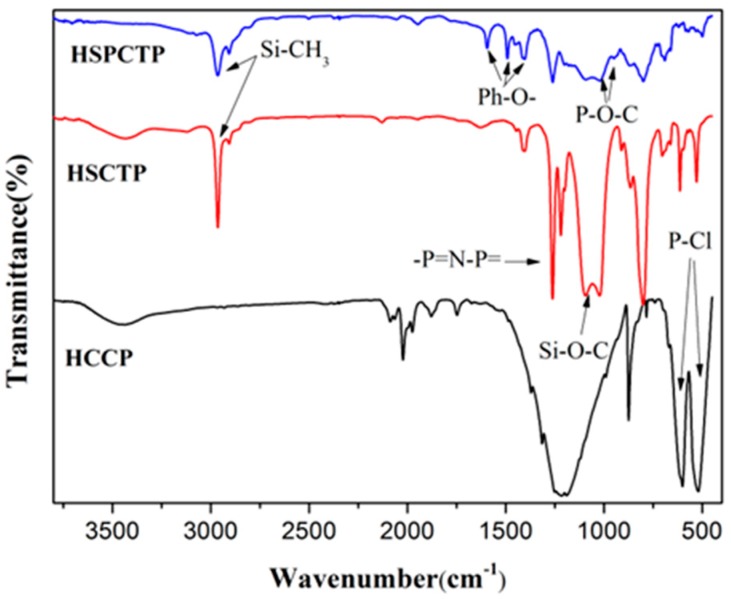
FTIR spectra of HCCP, HSCTP and HSPCTP.

**Figure 2:**
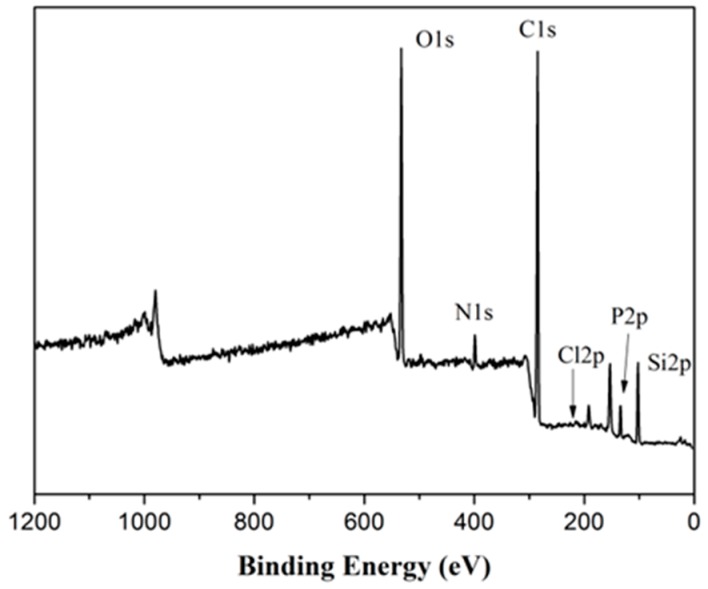
The XPS spectrum of the synthesized HSPCTP.

Figure 1 illustrates the FTIR spectra of HCCP, HSCTP and HSPCTP. All the specimens observed reached a characteristic P=N absorption peak at around 1250 cm^−1^, which indicates that the structure of the phosphazene ring is not destroyed after the incorporation of silicone oil and phenol. Compared with HCCP, the spectrum of HSCTP reached two new peaks at 2960 cm^−1^ and 1000~1130 cm^−1^, which belong to the Si–CH_3_ and Si–O bonds from dihydroxypropyl silicone oil, respectively. In other words, the silicone oil was successfully introduced into the phosphazene ring. Meanwhile, another two peaks appear at 1500 cm^−1^ and 970 cm^−1^ in the spectrum of HSCTP, which are attributed to the deforming vibration of the benzene ring skeleton and the stretching vibration of the P–O–C bond, respectively. This indicates that the partial chlorine atom in the phosphazene ring was replaced by the phenoxy group. Notably, the vibration peak (600 cm^−1^ and 520 cm^−1^) of the P–Cl bond almost disappears in the spectrum of HSCTP, suggesting that the chlorine was almost replaced by the silicone oil and phenoxy groups. This is further confirmed by the XPS spectrum, as shown in Figure 2, and the elemental data of HSPCTP are summarized in Table 1. It is found that the weight ratio of chlorine is as low as 0.63 wt%, which suggests that it can meet the requirements of RoHS (the Restriction of the use of certain hazardous substances in electrical and electronic equipment).

#### 3.1.2. Nuclear Magnetic Characterization

**Figure 3:**
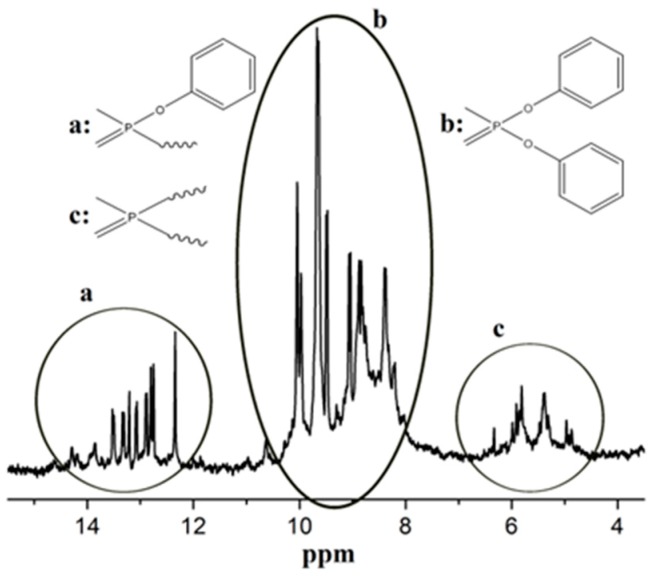
The ^31^P NMR spectrum of the synthesized HSPCTP.

**Figure 4:**
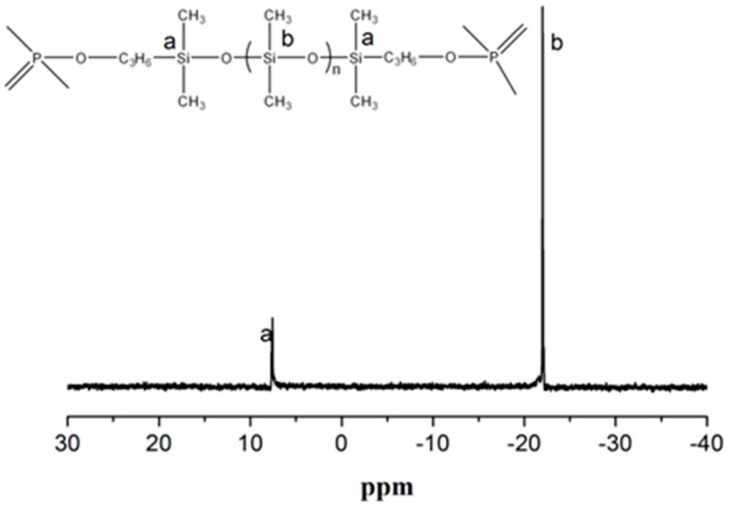
The ^29^Si NMR spectrum of the synthesized HSPCTP.

The chemical structure of the target product HSPCTP was further characterized via NMR including ^31^P and ^29^Si NMR, as shown in Figure 3 and Figure 4, respectively. For the ^31^P NMR spectrum, the peaks at 4.90–6.40 ppm and 8.00–10.53 ppm correspond to the phosphorus with two dihydroxypropyl silicone oil groups and two phenoxy groups, respectively. The peak at 12.30–14.35 ppm is attributed to the phosphorus with one phenoxy group and one dihydroxypropyl silicone oil group. In other words, the synthesized HSPCTP is a mixture of different groups. The ^29^Si NMR spectrum shows two peaks, which correspond to two different environments of silicon in the dihydroxypropyl silicone oil. The results indicate that the groups of dihydroxypropyl silicone oil were successfully connected to the hexachlorocyclotriphosphazene.

### 3.2. Effect of HSPCTP on the Flame Retardancy of PC-Based Blends

The flame retardancy of pure PC and its blends were measured in terms of LOI and UL-94 testing, and the experimental data are listed in Table 2. PTFE can prevent PC melt dripping and talc can strengthen the mechanical properties of PC. Both pure PC and PC/talc/PTFE specimens give 25% of LOI value and UL-94 V-2 rating due to the serious dripping. Notably, the incorporation of HSPCTP can improve the flame retardancy of the PC-based blends. In the case of 2 wt% HSPCTP loading level, the PC-based blend shows 26.2% of the LOI value and UL-94 V-1 rating without dripping. Furthermore, the incorporation of 3 wt% HSPCTP endows the PC-based blend with 28.4% of LOI and UL-94 V-0 rating without dripping. On the other hand, we calculated the chlorine content (W) of PC/HSPCTP3 with the formula shown in Equation 1 and found that the chlorine content of PC/HSPCTP3 is 0.017%, which is lower than that of RoHS.

(1) $$W=\left[\frac{m_{\left(Cl\right)}}{m_{\left(PC\right)}+m_{\left(Talc\right)}+m_{\left(PTFE\right)}+m_{\left(HSPCTP\right)}}\right]\times 100\%$$

### 3.3. Cone Calorimetry Analysis

The cone calorimeter has been widely used to evaluate the flammability and potential fire safety of polymer materials, which can reflect the flame-retardant properties of materials precisely [3,4]. Table 3 presents detailed information on the combustion behavior of pure PC and PC/HSPCTP3 composites obtained from the cone calorimeter tests at a heat flux of 50 kW/m^2^.

As shown Table 3, the time to ignition (TTI) of PC/HSPCTP3 composites decreases from 58 to 41 s. This reveals that the flame-retardant additive of HSPCTP not only decomposes ahead of time itself, but also promotes the PC matrix to degrade at a lower temperature, which contributes to charring earlier during combustion and improves the flame retardancy of PC. This phenomenon is in agreement with others reports [24,25,27].

**Figure 5:**
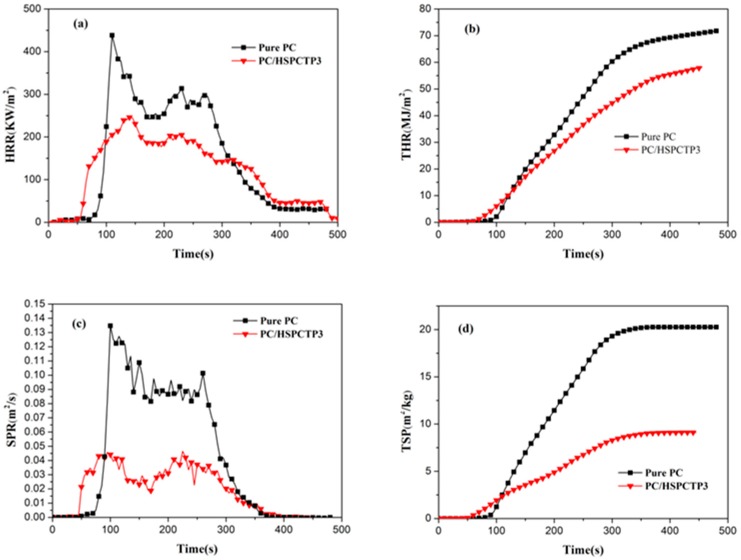
Heart release rate (HRR) (**a**), total heat release (THR) (**b**), smoke produce rate (SPR) (**c**) and total smoke production (TSP) (**d**) curves of pure PC and PC/HSPCTP3 composites.

The heat release rate (HRR) and the total heat release (THR) are important parameters to quantify the size of fire. Lower HRR and THR values show an effective flame retardancy. Figure 5 gives the HRR, the THR, the smoke produce rate (SPR) and the total smoke production (TSP) curves of pure PC and PC/HSPCTP3 composites. The results show that pure PC burns rapidly after ignition and the highest peak of HRR appears at 110 s, with a peak heat release rate (PHRR) of 438.38 kW/m^2^, as Figure 5a and Table 3 reveal. However, the highest HRR peak of PC/HSPCTP3 composites appears at 140 s with a PHRR of 245.90 kW/m^2^, which is later and lower than that of pure PC. This may be because the HSPCTP additive decomposes and promotes the degradation and charring of the PC matrix earlier. Besides, silicon oxide derivatives and phosphoric acid derivatives are produced by decomposition of HSPCTP, which promotes decomposition of PC [5,20,30,31]. Figure 5b and Table 3 show that the THR curve of PC/HSPCTP3 composites is lower than that of pure PC, and the THR value decreased from 71.56 to 59.11 MJ/m^2^. This is attributed to the fact that the PTFE formed a network structure after mixing and to the decomposition of HSPCTP, which contains phosphazene and hydroxypropyl silicone oil groups [32,33,34,35,36,37,38]. These results show that HSPCTP has a synergistic flame-retardant effect with PTFE, and that HSPCTP plays a major role in flame retardancy.

Figure 5c and Table 3 show that the SPR curve of pure PC presents a highest peak at 105 s with a value of 0.135 m^2^/s. However, the SPR curve of PC/HSPCTP3 composites appears to peak at 49 s, which is earlier than that of pure PC. This phenomenon indicates that HSPCTP decomposed first and produced CO_2_ and phosphoric acid. Besides, the SPR curve of PC/HSPCTP3 composites is lower than that of pure PC. This also indicates that HSPCTP promotes the formation of a dense carbon layer by PC, which suppresses combustion and decreases the release of a large quantity of smoke. From Figure 5d and Table 3, it is evident that the TSP value of PC/HSPCTP3 is 11.57 m^2^/kg, which is smaller than that of pure PC. This also shows that HSPCTP has a good smoke-suppressing effect on PC.

As Table 3 reveals, the average value of effective heat combustion (EHC) for PC/HSPCTP3 is 15.90 MJ/kg, which is smaller than that of pure PC. This phenomenon occurs because HSPCTP contains phosphazene and dihydroxypropyl silicone oil groups, which produce noncombustible gases, such as NH_3_, CO_2_, H_2_O and volatile phosphide during the combustion process [32,33,34,35,36,37,38]. Finally, the average value of HRR for PC/HSPCTP3 is 131.93 kW/m^2^, which is also smaller than pure PC. As discussed above, HSPCTP exhibits a high flame retardancy.

### 3.4. Morphology and EDS Analysis of Char Residue

**Figure 6:**
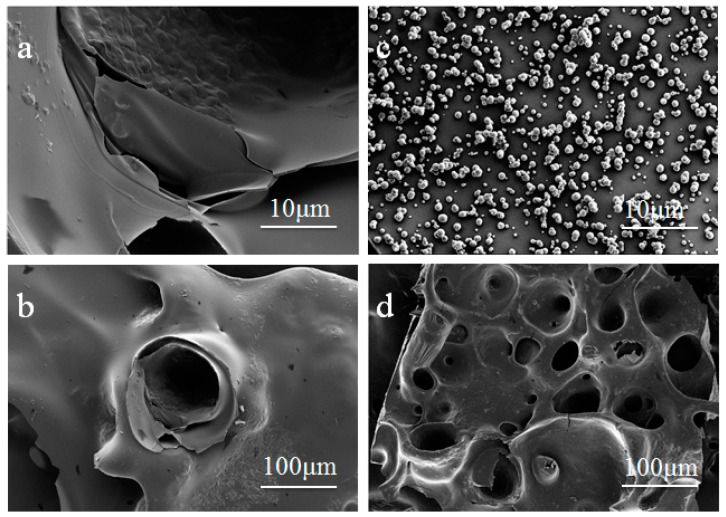
SEM images of the char residue of the PC/HSPCTP composites: outer surface of pure PC (**a**), PC/HSPCTP3 (**c**); inner surface of pure PC (**b**), PC/HSPCTP3 (**d**).

To investigate the relationship between the morphology of the char layers and the flame-retardant properties of the PC/HSPCTP composites, the char residues of the PC/HSPCTP composites are measured by SEM, as shown in Figure 6. It is evident that the outer surface of pure PC presents large holes due to insufficient char formation during combustion, as shown in Figure 6a; therefore, heat and flammable volatiles can penetrate the char layer and enter the flame zone. The inner surface has larges holes and cracks, as shown in Figure 6b, which lead to reduced flame retardancy. On the contrary, there is no hole observed on the outer surface of the PC/HSPCTP3 composite char layer, but there are many microspheres on the surface, as shown in Figure 6c. This is due to the decomposition of HSPCTP. These microspheres block the pores and promote the formation of a dense and regular carbon layer, which improves the flame retardancy of PC. Another reason is that the silica derivatives react with the PC chain to produce a cross-linked structure, which also promotes the formation of a compact carbon layer. In addition, phosphorus, nitrogen and benzene ring structures can also promote the formation of a regular carbon layer [16,25,26,27,28,29,30,39,40]. The inner surface, shown in Figure 6d, has more holes and microspheres. This phenomenon may be due to a large amount of inert gases, such as carbon dioxide, being decomposed by HSPCTP [15,16,25,26,27,28,29,30,39,40]. 

**Figure 7:**
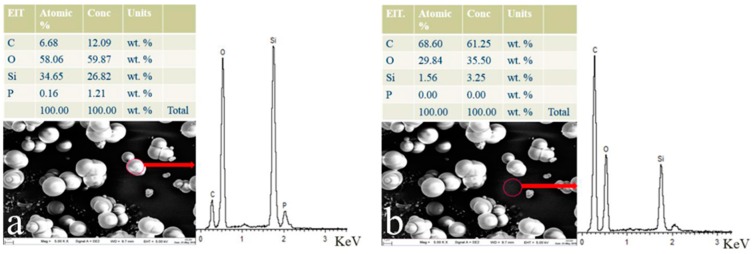
EDS results of the surface for PC/HSPCTP3 composites.

To further investigate the quality of the residual char, the composition of the char layer was investigated by the scanning electron microscopy/energy dispersive X-ray spectrometry (SEM/EDS). It was found that microspheres on the char surface of the PC/HSPCTP3 blend contain the elements of C, O, P and Si, as shown in Figure 7a. This suggests that the microspheres may be silica microspheres, which block the pores and promote the formation of a dense and regular carbon layer. However, the non-microsphere part of the carbon layer contains the elements of C, O and Si, as shown in Figure 7b, which indicates that the silica derivatives react with the PC chain to produce cross-linked structures, and also promote the formation of a compact carbon layer [15,16,25,27,28,29,30,39,40]. Although the peak corresponding to phosphorus in Figure 7b contains a small portion, it is lower than the lower limit of instrument detection.

### 3.5. FTIR Spectra of Residual Char

**Figure 8:**
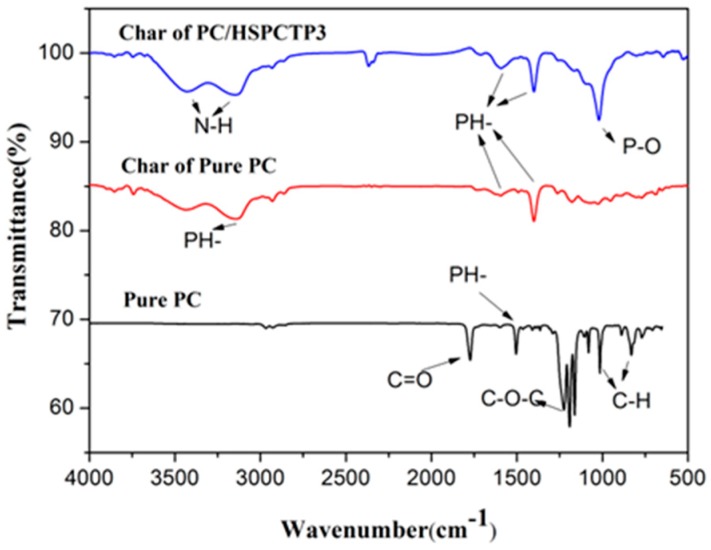
FTIR spectrum of the residual char of Pure PC, PC/HSPCTP3 composites obtained after the cone calorimeter test.

In order to further investigate the formation of the intumescent char layer, the residual char of pure PC and PC/HSPCTP3 composites was examined by FTIR spectra, as revealed in Figure 8. The peak of pure PC at 1761 cm^−1^ belongs to the ester bond (–COO–), which is a part of the PC chain structure. The multiple peaks around 760 cm^−1^ correspond to the characteristic of the para-substituted benzene ring. The multiple peaks around 1200 cm^−1^ correspond to the C–O bonds. These prove the integrity of the PC chain structure. However, the peak of pure PC disappears at 1761 cm^−1^ and decreases at around 1500 cm^−1^, 1200 cm^−1^ and 760 cm^−1^ or even disappears. This phenomenon indicates that the PC chain structure was destroyed after burning. Besides, new peaks around 3405 cm^−1^ and 3098 cm^−1^ appeared, which correspond to the hydroxyl group and the C–H bonds on phenol, respectively. The peaks at 2873 cm^−1^ and 2901 cm^−1^ correspond to the aliphatic C–H bonds. The peaks around 1750 cm^−1^, 1300 cm^−1^ and 1100 cm^−1^ correspond to the aromatic ester groups. These also indicate that PC decomposes to produce small molecules after burning.

Compared with pure PC, the peaks at 3405 cm^−1^ and 3098 cm^−1^ of the residual char for PC/HSPCTP3 became stronger. This was caused by the degradation of HSPCTP, which reacted with PC and produced aliphatic amines (N–H, 3405 cm^−1^) and aromatic amines (N–H, 3098 cm^−1^). The peaks at 2370 cm^−1^ and 2365 cm^−1^ belong to P–H bonds, which indicates that compounds containing phosphorus appeared. The peaks at 1460 and 1580 cm^−1^ are assigned to the remaining aromatic rings after combustion [8,9,10,11,12,13,14,15,16,25,27,28,29,30,39,40]. The peak at 1196 cm^−1^ is attributed to the stretching vibration of P–N bonds in phosphazene, which provides direct evidence for the phosphazene structure with excellent thermal stability existing in the residual char [10,11,12,13,14,15,16,26,27,28,29,30,39,40]. The peak at 1110 cm^−1^ is ascribed to the stretching vibration of PO_2_/PO_3_ in phosphate carbon complexes and aliphatic Si–O–C and Si–C bonds, and the peak at 912 cm^−1^ belongs to the stretching vibration of the P–O–P bond [39,40]. These indicate that polyphosphoric acid and silicon-containing compounds appeared during the thermal degradation of PC. The formed acid can promote the formation of the carbonaceous char by carbonization. The silicon-containing compounds also react with the PC matrix to produce a cross-linked structure, which improves drip resistance and carbon layer formation.

### 3.6. FTIR Spectra of Residual Char

**Figure 9:**
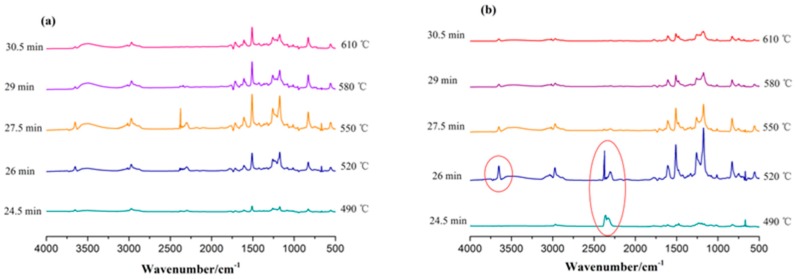
TG-IR spectra of pure PC (**a**) and PC/HSPCTP3 (**b**) at different decomposition temperatures.

To understand the flame-retardant effect of HSPCTP on PC, the TG-IR of pure PC and PC/HSPCTP3 were investigated, as shown in Figure 9. It is clear that both the curves of pure PC and PC/HSPCTP3 show characteristic bands of water (3650 cm^−1^, 1259 cm^−1^, O–H bond) [41,42], aliphatic compounds (2972 cm^−1^, 1248 cm^−1^, 748 cm^−1^) [31,43], carbon dioxide (2300–2400 cm^−1^) [31,43], compounds containing aromatic rings (1610 cm^−1^, 1505 cm^−1^) [31,43] and nitrogen compounds (1200 cm^−1^) [41]. The characteristic bands of the two samples are similar.

Moreover, it is also observed that the pure PC and other composites start to produce gas products at 24.5 min at a temperature of 590 °C. Pure PC continues to generate gas from 490 °C to 610 °C. However, PC/HSPCTP3 composites release a lot of gas products at 26 min with the temperature at 520 °C. As the temperature increases, the amount of gas generation reduces significantly. It can be concluded that HSPCTP can promote decomposition to produce a large amount of inert gas to achieve a flame-retardant effect quickly.

### 3.7. Flame-Retardant Mechanisms of PC/HSPCTP Blends

**Figure 10:**
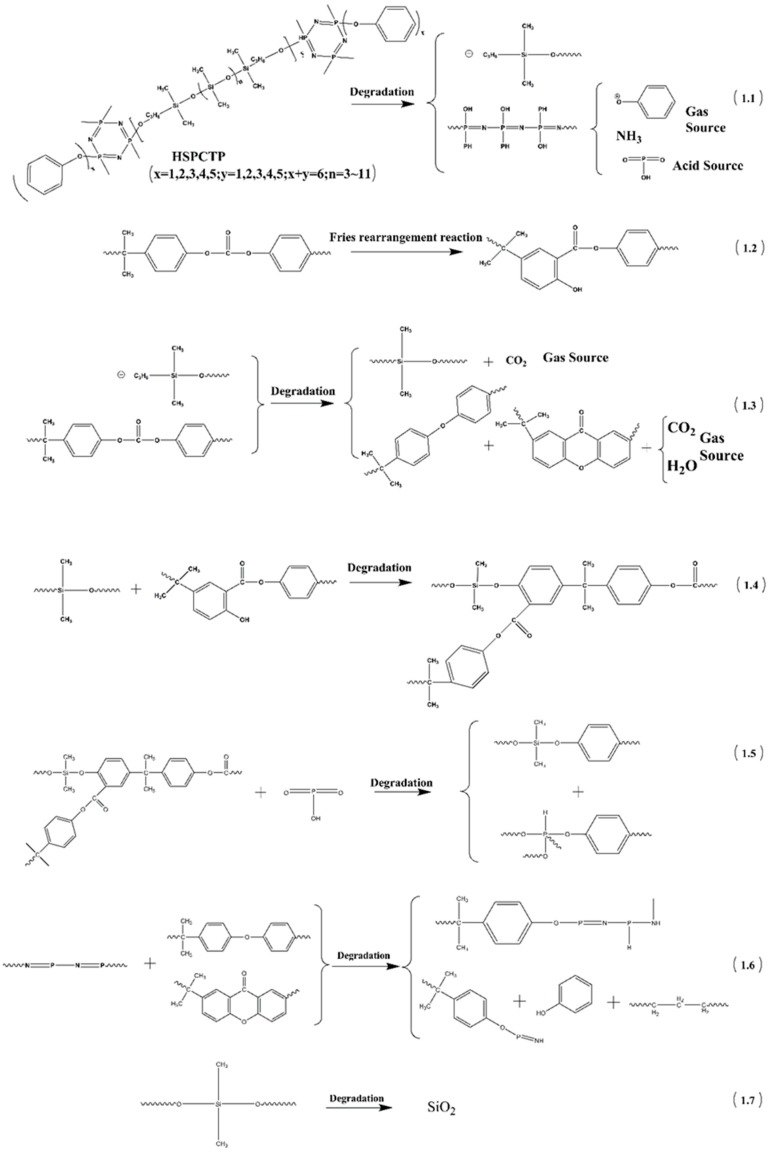
Schematic illustration of the flame-retardant mechanism of PC/HSPCTP composites.

As discussed above, HSPCTP is an efficient flame retardant for PC, which involves both a gas phase and a solid phase flame-retardant effect. As shown in Figure 10, the main degradation process is separated into three stages. In the first degradation stage, HSPCTP decomposed into silicone derivatives and a phosphazene ring-opened compound (chemical reaction 1.1). In this case, the phosphazene ring-opened compound further decomposed into phenoxy radical (PH–O·), ammonia (gas source) and metaphosphoric acid (acid source) [39,40]. Moreover, the phosphazene ring-opened compound is prone to generating phosphorus-based radicals (PO·, PO_2_·, etc.), which could manifest as radical scavengers to trap the H· and HO· for combustion reactions in the gas zone; it is believed that these radical scavengers are beneficial to flame retardancy [40]. With increasing temperature, the Fries rearrangement reaction of PC chains occurs (chemical reaction 1.2) [31]. This reaction can accelerate cross-linking and the carbon formation of PC.

In the second degradation, the metaphosphoric acid accelerated the dehydration of PC, and the silicone derivatives decomposed into other small molecule silicone derivatives and carbon dioxide (chemical reaction 1.3) [6,7,8,9,10,11,12,13,14,15,31]. The reactions 1.1 and 1.3 produced inert gases to promote flame retardancy.

In the last stage, silicon-containing groups attacked the hydroxyl groups formed after the PC rearrangement reaction to form cross-linked carbon-forming silyl ether structures, which promoted the formation of dense, regular carbon layers (chemical reaction 1.4) [5,6,7,8,9,10,11,12,13,14,15,16,17,18,31]. The silicon-containing compounds also reacted with the PC matrix to produce a cross-linked structure, which improved drip resistance and carbon layer formation. Besides, the formed acid can react with the PC chains and promote the formation of cross-linked structures containing phosphorus (chemical reaction 1.5). Undecomposed cyclotriphosphazene reacted with short-chain molecules produced by the decomposition of PC, and then produced phenol, nitrogen-containing compounds, phosphorus-containing compounds and aliphatic compounds (chemical reaction 1.6). Some parts of silicon oil groups also turned out to be SiO_2_ (chemical reaction 1.7).

## 4. Conclusions

A novel flame retardant, HSPCTP, with phenoxy and hydroxypropyl silicone oil groups, was designed and incorporated into PC to improve its flame retardancy. Combining the advantages of cyclotriphosphazene and silicon compounds, PC composites passed UL-94 V-0 rating with only 3 wt% HSPCTP. This study found that HSPCTP stimulated PC matrix decomposition and char formation ahead of time and enhanced the char yield and the thermal stability of PC/HSPCTP composites at high temperature. Furthermore, HSPCTP also promoted the formation of a uniform, sealing, intumescent and continuous char layer with high thermal stability due to the existence of an abundant aromatic structure, phosphazene and hydroxypropyl silicone oil groups in the HSPCTP additive. The formed char layer prevented heat transmission and diffusion, limited the production of combustible gases, inhibited the emission of smoke and then led to the reduction of the heat release rate and the smoke produce rate. On the other hand, hydroxypropyl silicone oil groups generated a large amount of inert gases, such as carbon dioxide, at a high temperature, which diluted flammable and combustion-supporting gas. In other words, HSPCTP showed both gas-phase and solid-phase flame-retardation. As a result, the introduction of the prepared HSPCTP flame retardant additive to PC led to a higher flame-retardant efficiency and thermal stability.

## Figures and Tables

**Table 1:** Elemental data of HSPCTP.

Sample	Peak BE (eV)	Area (P)	Atom (%)	Quality (wt%)
**C 1s**	281.95	16,394.23	58.49	44.10
**N 1s**	395.35	1459.72	2.93	2.58
**Cl 2p**	197.65	179.06	0.28	0.63
**Si 2p**	99.1	3242.93	13.99	24.62
**P 2p**	131.14	1305.92	3.87	7.54
**O 1s**	529.59	16,307.59	20.45	20.54
**Total**			100.01	100.01

**Table 2:** Flame retardancy of the PC/HSPCTP blends.

Sample	PC wt%	Talc wt%	PTFE wt%	HSPCTP wt%	UL-94 Rating (1.8 mm)	Dripping or Not	LOI/%
Pure PC	100	0	0	0	V-2	Yes	25
PC-HSPCTP0	100	5	0.5	0	V-2	Yes	25
PC-HSPCTP2	100	5	0.5	2	V-1	No	26.2
PC-HSPCTP3	100	5	0.5	3	V-0	No	28.4

**Table 3:** Cone calorimeter data for pure PC and PC/HSPCTP3 composites.

Sample	Pure PC	PC/HSPCTP3
TTI(s)	58	41
Peak-HRR(kW/m^2^)	438.38	245.90
t _Peak-HRR_(s)	110	140
THR(MJ/m^2^)	71.56	59.11
Peak-SPR(m^2^/s)	0.135	0.045
t _Peak-SPR_(s)	105	98
TSP(m^2^/kg)	20.25	11.57
av-EHC(MJ/kg)	23.82	15.90
av-HRR(kW/m^2^)	170.26	131.93
